# *Staphylococcus haemolyticus* epididymo-orchitis and bacteraemia: a case report

**DOI:** 10.1099/jmmcr.0.005157

**Published:** 2018-07-05

**Authors:** Christina Pindar, Roberto A. Viau

**Affiliations:** ^1^​ Case Western Reserve, University School of Medicine, Cleveland, OH 44106, USA; ^2^​ Department of Medicine, Case Western Reserve University, Louis Stokes Cleveland VA Medical Center, Cleveland, OH 44106, USA

**Keywords:** epididymitis, orchitis, epididymo-orchitis, bacteraemia, *Staphylococcus haemolyticus*

## Abstract

**Introduction:**

Although more often recognized as a culprit in female urinary tract infection, coagulase-negative staphylococci (CoNS) can cause severe genitourinary infections in men. While positive blood cultures with CoNS are usually thought to be contaminants, in the setting of a severe genito-urinary infection they can represent true infection.

**Case presentation:**

We present the case of a 70-year-old male without a central venous catheter or urinary catheter who developed *Staphylococcus haemolyticus* bloodstream infection secondary to epididymo-orchitis.

**Conclusion:**

This case highlights the importance of prompt recognition of serious CoNS infections, including bacteraemia, in the setting of CoNS genitourinary tract infections.

## Introduction

Coagulase-negative staphylococci (CoNS) are known pathogens of the genitourinary system, often implicated in urinary tract infections (UTIs). A surveillance study conducted in Japan found *Staphylococcus saprophyticus* to be the most common Gram-positive pathogen, second to *Escherichia coli* as the most common overall cause for female UTI [1]. In a cross-sectional study of emergency room UTI diagnoses in a paediatric population, *S. saprophyticus* was identified as the third most common pathogen, although it was the second most common pathogen in older children. Another CoNS, *Staphylococcus warneri*, was identified as the causative agent for two UTIs. *S. saprophyticus* appeared in 11 of the female UTI specimens, but only 1 of the male specimens, a difference that was not statistically significant [2]. These findings highlight that, despite its commonality in female patients, *S. saprophyticus* is very rarely implicated in male genitourinary infections. However, this pathogen cannot be entirely excluded when considering male UTI pathogens, especially in older male patients. A Swedish study noted that 6.1 % of *S. saprophyticus* isolates came from male urine samples and another study similarly reported *S. saprophyticus* isolates from male UTI samples [3, 4].

CoNS have been frequently considered contaminants when identified in blood cultures [5, 6]. Beyond being implicated in localized infections, such as UTIs, they can cause systemic infections such as sepsis and endocarditis, and are notably resistant to many antibiotics [5–12]. In one study of *S. saprophyticus* native valve endocarditis, the infection was noted to originate in the urinary tract; thus, demonstrating the ability of CoNS genitourinary infections to result in systemic infection [13]. We present the case of a 70-year-old male without a central venous catheter or urinary catheter who developed *Staphylococcus haemolyticus* bloodstream infection secondary to epididymo-orchitis.

## Case report

A 70-year-old sexually active African American male with a past medical history of untreated hepatitis C, erectile dysfunction treated with vardenafil, hypothyroidism and hypertension presented to the Louis Stokes Department of Veterans Affairs Medical Center emergency department with a 1 week history of dull, aching pain in the right testicle that had increased in severity the day prior to admission after doing yard work. He denied erythema or pain of the contralateral testicle or penile discharge. He reported haematospermia three times in the preceeding 4 weeks. He reported urinary urgency and frequency the night prior to admission, but had no dysuria, difficulty initiating a stream or haematuria. He also denied pelvic pain or rashes. He had protected intercourse regularly with a healthy single female partner. He reported a remote history of unspecified sexually transmitted infection (STI) that was treated.

On admission, vital signs were relevant for tachycardia to 107 beats min^−1^. On physical examination, his right testicle was swollen and tender, but non-erythematous with no abnormal masses. His prostate was non-tender and non-enlarged. He had no inguinal lymphadenopathy, no inguinal hernias and no penile discharge. His examination was otherwise unremarkable. His complete blood count was notable for a white blood cell (WBC) count of 40 000 with neutrophil predominance (89.5 %). Prostatic Specific Antigen (PSA) was 6.4 ng ml^−1^, elevated from 1.0 ng ml^−1^ 1 year prior. Urine culture grew 10 000 c.f.u. normal urogenital flora, while urinalysis showed 13 WBCs per high power field small leukocyte esterase and negative nitrites. PCR identification tests for chlamydia and gonorrhoea from a first-voided urine specimen were negative. Testicular ultrasound showed right epididymitis and orchitis with bilateral hydroceles. The patient developed fever to 39 °C during the admission.

The patient received ceftriaxone, 1 g (to cover community acquired Gram-negative rods and *Neisseria gonorrhoeae*), and azithromycin, 1 g (to provide double coverage against *N. gonorrhoeae* as well as *Chlamydia trachomatis*). Coverage of STIs was given due to a higher STI risk in the veteran population [14]. The antibiotic regimen was later changed to ciprofloxacin (400 mg iv every 12 h), assuming epididymo-orchitis secondary to enteric organisms. Despite antibiotics, the patient remained febrile. Blood cultures turned positive on hospital day 2, with both sets showing Gram-positive cocci in clusters on Gram staining. The antibiotics were changed to vancomycin (dose adjusted for trough level of 15 mg dl^−1^) and ceftriaxone (1 g every 12 h), and the fever resolved, while the testicular swelling persisted. A chest x-ray and computed tomographyscan of the abdomen and pelvis were unremarkable. Meanwhile, the WBC count down trended to 22 000 and resolved after 48 h antibiotic therapy. The final microbiology result was of *S. haemolyticus* sensitive to vancomycin. The patient received intravenous vancomycin for 2 weeks, followed by oral clindamycin (300 mg every 6 h) for 1 week. Repeat blood cultures were negative. A repeat testicular ultrasound prior to discharge demonstrated continued evidence of right epididymo-orchitis, which resolved on follow up ultrasound 3 weeks after discharge.

## Discussion

Most often, epididymo-orchitis is due to Gram-negative rods, such as *E. coli*. These bacteria originate from the gastrointestinal tract, infect the urinary bladder, prostate or urethra, and reflux to the epididymis. Given this pathophysiology, risk factors for epididymo-orchitis include UTIs, bacterial prostatitis, prostatic obstruction, urinary stasis, instrumentation/catheterization of the bladder and congenital anomalies in the genitourinary tract. Alternatively, in sexually active men, *C. trachomatis* and *N. gonorrhoeae* are frequent culprits [15].

CoNS are alternate causes of UTIs. One study reported that while *S. saprophyticus* and *Staphylococcus epidermidis* were isolated from 81 % of female UTIs, *S. epidermidis*, *S. warneri* and *S. haemolyticus* were isolated from 87 % of male UTIs [7]. *S. saprophyticus* has been found to colonize both the gastrointestinal and urogenital tracts, most commonly the rectum, of 6.9 % healthy female subjects and is found in skin flora [4, 16, 17]. *S. haemolyticus* is noted to be found colonizing not only the skin, but also the urethra and periurethra of both males and females [9, 17]. These pathogens can cause genitourinary infections in both anatomically normal males and females, and those with urogenital abnormalities [4, 16, 18, 19]. CoNS, particularly *S. epidermidis*, also form biofilms infecting patients with indwelling urinary catheters [11]. Therefore, the prevalence of CoNS infections continues to rise as healthcare professionals continue to utilize invasive devices that are susceptible to colonization by CoNS [6].

Our patient presented with epididymo-orchitis in the setting of *S. haemolyticus* bacteraemia. Surprisingly, he had a benign urinalysis and a urine culture showing normal urogenital flora, although this may be similar to findings that show that *S. saprophyticus* is found in low numbers in the bladder and voided urine of patients with *S. saprophyticus* cystitis [17]. It is possible that this patient had *S. haemolyticus* colonization below the 100 000 c.f.u. threshold that would have prompted the microbiology laboratory to attempt speciation of the isolate. Furthermore, our clinical laboratory reported ‘urogenital flora’ that does include CoNS.

Although CoNS are recognized as potential culprits of both UTIs and bacteraemia, this appears to only be the second report of *S. haemolyticus* genitourinary infection [19]. The lack of typical risk factors for CoNS bacteraemia, such as the presence of an indwelling vascular device, makes this case particularly interesting. There are two possible explanations for this patient’s presentation. It is possible that he first developed bacteraemia, which then seeded the epididymis. However, since his presenting complaint was scrotal swelling and prior haematospermia, in the absence of systemic signs and symptoms, it is more likely that he first developed the epididymo-orchitis that then progressed to bacteraemia. It is worth noting that infections with CoNS differ from those with *Staphylococcus aureus* in that they are more indolent, with subacute or chronic presentations that rarely become truly fulminant [5].

*S. haemolyticus* has been identified as a frequent cause of bacteraemia, being cited in one study as the most common bloodstream pathogen in likely or possible bloodstream infections. A majority of these infections were associated with foreign bodies [20]. Another study found that *S. haemolyticus* was the second most prevalent bloodstream pathogen after *S. epidermidis*. It found that central venous catheters, prior antibiotic therapy, more than one positive blood culture and ICU admission were significantly associated with CoNS bloodstream infection [11]. CoNS bacteraemia is most frequently thought to be related to inoculation by central venous catheters and, therefore, from a patient’s skin. However, a previously proposed hypothesis suggests that the gut is the primary contributor to CoNS bacteraemia due to mucosal colonization [8].

It is likely that our patient’s epididymo-orchitis progressed in the same way as most cases do: bacteria, originating from the gastrointestinal tract, infected the urinary bladder or urethra, and refluxed to the epididymis. As it is well known, CoNS are not uncommon inhabitants of the gastrointestinal or genitourinary tracts, and have been found colonizing the urethra and periurethra [19]; thus, their role as genitourinary pathogens should be recognized. Our case highlights the importance of prompt recognition of CoNS bloodstream infections in the right clinical setting. Clinicians should not ignore the possibility of serious CoNS infections stemming from the genitourinary tract.
